# Diagnosis of vaccination rooms in Brazilian primary health care centers taking part in the PlanificaSUS project, 2019

**DOI:** 10.1590/S2237-96222022000200016

**Published:** 2022-07-08

**Authors:** Evelyn Lima de Souza, Ilana Eshriqui, Eliana Tiemi Masuda, Daiana Bonfim, Rubia Pereira Barra, Márcio Anderson Cardozo Paresque

**Affiliations:** 1 Hospital Israelita Albert Einstein, Centro de Estudos, Pesquisa e Prática em APS e Redes, São Paulo, SP, Brazil; 2 Centro Colaborador da Planificação da Atenção à Saúde, Uberlândia, MG, Brazil

**Keywords:** Vaccination, Primary Health Care, Immunization Programs, Quality of Health Care

## Abstract

**Objective:**

To describe the diagnosis of vaccination rooms in primary healthcare centers in Brazil.

**Methods:**

This was a cross-sectional study with secondary data of convenience sampling comprised of 25 rooms. Results of a checklist adapted from the Vaccine Room Supervision Tool of the National Immunization Program in 2019 regarding the dimensions ‘general organization’, ‘general aspects’, ‘technical procedures’, ‘cold chain’, ‘information system’, ‘adverse events following vaccination’, ‘special immunobiological agents’, ‘epidemiological surveillance’ and ‘health education’, were used. Percentages of scores, both overall and by dimensions were described in median, interquartile range, minimum and maximum values.

**Results:**

The overall median was 77.1%, higher for ‘health education’ (100.0%) and ‘cold chain’ (86.7%), and lower for ‘special immunobiological agents’ (50.0%) and ‘general organization’ (58.3%).

**Conclusion:**

Using the checklist enabled the diagnosis in different macro-regions, inter- and intra-regional differences were found in the dimensions, and positive results and opportunities for improvement in the general plan.

**Study contributions**

**Main results**

A greater strengthening in the dimensions ‘health education’ and ‘cold chain’ stands out, with scores of at least 87%; ‘special immunobiological agents’ and ‘general organization’ obtained median scores lower than 60%. It can be seen inter- and intra-regional heterogeneity.

**Implications for services**

The checklist used proved to be easily applicable for the diagnosis, identification of opportunities for improvement, and vaccination room monitoring in the five regions of Brazil.

**Perspectives**

We highlight the need to update the checklist and strengthen the actions in dimensions with unfavorable results, especially in some regions of the country, taking into consideration the local needs.

## Introduction

The National Immunization Program (PNI) coordinates immunization activities in Brazil and has contributed to the history of high vaccination coverage, in addition to ensuring universal and free access to immunobiological agents.^1^ However, currently there has been a decrease and heterogeneity in vaccination coverage in the country.^2^ ^-^ ^5^ Given the reemergence of vaccine-preventable diseases,^7^ ^-^ ^10^ the need to strengthen the evaluation and organization actions of the PNI and health services stands out.^6^

Regarding the so-called Health Care Planning, a methodology that aims at the organization and integration of services in health care networks,^11^ ^,^ ^12^ the strengthening of these actions includes storage organization, logistics, dose administration and local coverage monitoring, contributing to the adequacy of vaccination rooms to meet the standards recommended by the PNI.^6^

Few studies address the diagnosis of vaccination rooms at the local level and there are no studies addressing this subject at the national level.^13^ ^-^ ^17^ Therefore, the objective of this Research Note was to describe the diagnosis of vaccination rooms in Primary Healthcare Centers (PHC) that executed the Health Care Planning in 2019.

## Methods

### Study design

This was a cross-sectional study based on secondary data from a convenience sampling of vaccination rooms distributed over the five Brazilian macro-regions.

### Setting

The universe of this study was comprised of 26 PHCs, each of them was located in a regional health department in 20 Federative Units. These PHCs were selected as a model for the execution of the Planning process in the first stage of the PlanificaSUS project, whose official name is ‘Organization of Specialized Outpatient Care in Network with Primary Health Care’,^18^ carried out between 2018 and 2020.

### Participants

Among the 26 PHCs selected, 25 had a vaccination room, therefore they were eligible to take part in this study.

### Variables

Variables of interest:

- Overall vaccination room score (%): (sum of checklist items that were implemented/number of checklist items) x 100
- Vaccination room score by dimension (%): (sum of dimension items that were implemented/number of dimension items) x 100
- Location of PHCs according to the five Brazilian macro-regions.

### Data source and measurement

Vaccine Room Supervision Tool, developed by the PNI^19^ and adapted as a checklist by means of Microsoft Excel Spreadsheet, was used during PlanificaSUS activities aiming at performing the diagnosis and organizing vaccination rooms. The nursing professional who was responsible for the vaccination room filled out the checklist, pointing out whether or not the items were implemented (yes; no). PlanificaSUS tutor of the respective PHC was responsible for inserting the completed checklist file, in Excel, into the project platform (e-Planifica), where it was stored for consultation. This study used the files available in e-Planifica.

The checklist is comprised of 131 items, subdivided into nine dimensions (Box 1). The diagnosis was made by analyzing continuous measurements of the scores, both overall score and by dimension, as detailed in statistical methods.

**Box 1:** Dimensions and items of the diagnostic checklist of vaccination rooms

**Dimension 1: General organization**
The municipality has a vaccine coordinator
The municipal vaccine coordinator conducts periodic supervision of the vaccination room, verifying the standard procedures, compliance with indicators and training for professionals
The primary healthcare center has a nurse responsible for the vaccination room
The vaccination room provides services throughout the unit opening hours, including lunch time, every day
All vaccines from the current schedule of the National Immunization Program are administered during the entire period in which the vaccination room is open
There are technical standards related to vaccination and they are made available to all professionals: vaccine administration protocols; cold chain; epidemiological surveillance of adverse events; reference center for special immunobiological agents; and personnel training in the vaccination room
All professionals are aware of the technical standards
The set of standard operating procedures for the vaccination room is implemented and updated by the team, according to the current standards of epidemiological and health surveillance
All nurses and nursing technicians/assistants who work and are responsible for the vaccination process know the standard operating procedures for vaccination
All nurses and nursing technicians/assistants who work and are responsible for the vaccination process are up to date on vaccination and immunization procedures for the population in the coverage area
Specific training sessions are periodically carried out for all professionals who work in the vaccination room
The nurse in charge periodically supervises all procedures performed by the nursing technicians/ assistants who work in the vaccination room
**Dimension 2: General aspects of the vaccination room**
The room is exclusively used for vaccination
The room is easily accessible to the population
The room is properly identified
The physical area of the vaccination room meets the standards recommended by the General Coordination of the National Immunization Program/Agência Nacional de Vigilância Sanitária
Light-colored, waterproof, and easy-to-clean walls
Resistant and non-slip floor
Waterproof and easy-to-clean floor
The room has an easy-to-clean sink with a tap and a countertop
The room has adequate protection against direct sunlight
The room has adequate lighting
The room has adequate ventilation
The room is in ideal preservation conditions
The room is in ideal cleaning conditions
General cleaning (walls, ceiling, etc.) is done every 15 days
The room temperature is maintained between 18º C and 20º C
There are no decorative objects in the room (paintings, vases, etc.)
The furniture has a good functional distribution
Printed matters and information materials are organized
Syringes and needles of daily use are properly packed (in clean and capped containers)
Syringes and needles in the stock are packed in sealed packages and in place with no humidity
The room has dispenser with liquid soap and alcohol
The room has a medical examination table (stretcher) for vaccination, with a waterproof mattress or similar and protected with disposable material
The room has a chair to accommodate the user during his/her vaccination
**Dimension 3: Technical procedures**
Age and interval between doses are checked, at each vaccine administration
Adverse events from previous doses are investigated, at each vaccine administration
Temporary or permanent contraindication to the indicated vaccine is evaluated, at each vaccine administration
The technician in charge advises the user or guardian about the vaccine to be administered
The technician in charge instructs the user or guardian about the record of scheduling
The expiration date of the vaccine is observed, at each vaccine administration
Vaccine preparation is performed according to technical standards
The nurse responsible for the vaccination room periodically supervises the preparation of the vaccine performed by the various technicians of the team
Date and time of bottle opening are properly recorded
The expiration date after the bottle opening is observed
The vaccine administration technique follows the defined standards
The nurse responsible for the vaccination room periodically supervises the vaccine administration performed by the various technicians of the team
Perforating and cutting materials are packed according to the biosafety standards, in a container used to pack perforating and cutting materials
Vaccines with live microorganisms are treated before disposal, according to biosafety standards
Active search for susceptible individuals among users in the coverage area is conducted
Control cards (mirror cards) are used for vaccination in children
Control cards (mirror cards) are used for vaccination in adolescents
Control cards (mirror cards) are used for adult vaccination
Control cards (mirror cards) are used for older adult vaccination
Control cards (mirror cards) are used for vaccination in pregnant women
The control cards are organized by return date
Active search of defaulters is conducted
The number of vaccines is sufficient to meet the demand of the population
There is a vaccine stock management in the unit
The number of syringes and needles is sufficient to meet the demand
Expiration date of syringes and needles is observed
Different types of waste are packed separately
Final destination of the waste is in accordance with the norms of health surveillance
**Dimension 4: Cold chain**
Switches are available for the exclusive use of each equipment
On the electrical distribution box there is a warning not to turn off the vaccination room circuit breaker
The refrigerator is exclusively used for immunobiological agents
The capacity of the refrigerator is equal to or greater than 280 liters
The refrigerator is in good condition
The refrigerator is an ideal state of cleanliness
The refrigerator is away from heat sources, out of direct sunlight and at least 20 cm from the wall
There is a maximum and minimum thermometer and/or an extension cable in the refrigerator
Recyclable ice coils are kept in the evaporator in the recommended quantity
The refrigerator has a water drip tray
On the first shelf of the refrigerator, in perforated trays, only the vaccines that can be submitted to negative temperature are kept
On the second shelf of the refrigerator, in perforated trays, only the vaccines that cannot be submitted to negative temperature are kept
On the third shelf of the refrigerator, vaccine stocks, serum and diluents are stored
Immunobiological agents are organized by type, batch and expiration date
Distance between immunobiological agents and the walls of the refrigerator is kept in order to allow air circulation
Dyeing water bottles are kept in the entire lower internal space of the refrigerator
There is some material stored in the refrigerator inner door panel
The correct reading and recording of the temperature at the beginning and end of the workday is routinely conducted
Daily Temperature Control Chart is displayed in a visible place
Defrosting and cleaning of the refrigerator is performed every 15 days or when the ice layer reaches 1.0 cm
There is a preventive and/or corrective maintenance program for the vaccination room refrigerator
The service has a cooler box (polyurethane and/or expanded polystyrene - Styrofoam) or other equipment for daily use, in sufficient number to meet routine activities
The service has sufficient number of recyclable ice coils to meet routine activities
The service has a sufficient number of maximum and minimum thermometers and extension cables to meet routine activities
The service has sufficient number of polyvinyl chloride (PVC) tapes/crepe tapes to meet routine activities
The recyclable ice coils are stored in the organization of the cooler box
The temperature of the cooler boxes or daily use equipment is monitored
When, for any reason, the immunobiological agents are submitted to temperatures that are not recommended, the hierarchically superior authority is immediately communicated
When, for any reason, immunobiological agents are submitted to non-recommended temperatures, the evaluation form of the immunobiological agents under suspicion is filled out and sent to the hierarchically superior authority
When, for any reason, immunobiological agents are submitted to non-recommended temperatures, the vaccines under suspicion are kept at a temperature between +2ºC and +8ºC until the decision of the hierarchically superior authority
**Dimension 5: Information system**
The National Immunization Program Information System is implemented in the unit
All professionals know and routinely use the National Immunization Program Information System
The unit provides the forms and instruments for recording vaccination-related care
Child’s card
Adult card
Pregnant woman’s card
Older adult card
Daily Bulletin of Vaccine Doses Administered
Monthly Bulletin of Vaccine Doses Administered
Control Card (schedule)
Daily Temperature Control Chart
Adverse Event Investigation Form
Form for the Evaluation of Vaccines Under Suspicion
Monthly tendency of immunobiological agents
The technicians in charge know how to fill out the forms and tools properly
The nurse in charge periodically supervises the record of vaccination-related care
There is a routine monitoring and evaluation of vaccine indicators
The whole team takes part in the monitoring and evaluation moments
The vaccination coverage indicator is monitored
The dropout rate indicator is monitored
Professionals know and periodically discuss the available information
**Dimension 6: Adverse events following vaccination**
Information about the occurrence of adverse events is shared with all professionals
Professionals know the possible adverse events following vaccination for each type of vaccine
Professionals identify adverse events that should be referred for medical evaluation
Professionals report adverse events following vaccination
Investigation of occurrence of adverse events is carried out
**Dimension 7: Special immunobiological agents**
All professionals know about the existing Reference Center for Special Immunobiological Agents
Professionals know about the list of immunobiological agents available at the Reference Center for Special Immunobiological agents
Professionals are aware of the indications of these immunobiological agents
All professionals are aware of the flow to request these immunobiological agents and/or referrals for vaccination
**Dimension 8: Epidemiological surveillance**
Information about the occurrence of any vaccine-preventable disease cases in their area of coverage (measles, rubella, diphtheria, pertussis, tetanus, polio, rabies and others) is shared immediately with the whole team, generating the necessary alert
The team evaluates the information about the incidence of vaccine-preventable diseases, comparing it with vaccination coverage
The team takes part in transmission blocking vaccination, when indicated
The team reports suspected cases of diseases under epidemiological surveillance that come to their knowledge
**Dimension 9: Health education**
The team develops partnerships with social entities and community groups for dissemination and mobilization of the target population for immunization actions
The team uses educational programs in the primary healthcare center in order to suggest the theme of vaccination and immunization
All users attending the vaccination room are guided and informed about the importance of vaccines and compliance with the vaccination schedule
All staff members are informed about the available vaccines, the importance of being vaccinated, and referring users to the vaccination room

Source: Adapted from the Vaccine Room Supervision Tool.^19^

### Bias control

In order to reduce biases, the checklist was presented in a standard way by the project mentoring team, and the completion of all services occurred in the same operationalization phase of the Planning process.

### Study size

The study was comprised of 25 eligible rooms that had completed the checklist and inserted it into the e-Planifica.

### Statistical methods

A descriptive analysis of the scores, both overall and by dimension, of the vaccination rooms was performed, presenting median, interquartile range and minimum and maximum values. The items of the dimensions with the lowest median score were described. A scatter plot was used to verify the distributions of the rooms over the Brazilian macro-regions, according to the scores, both overall and by dimension.

Microsoft Excel and R, a statistical software package (v.4.1.0) were used.

### Ethical aspects

The study project was approved by the Research Ethics Committee of the Hospital Israelita Albert Einstein: Opinion CEP/Einstein No. 3,674,106, approved on October 22, 2019; Certificate of Submission for Ethical Appraisal (CAAE) No. 12395919.0.0000.0071.

## Results

A total of 25 rooms were studied, distributed over 19 states: 5 in the North region, 9 in the Northeast region, 3 in the Midwest region, 3 in the Southeast region and 5 in the South region.

The description of the scores, both overall and by dimension, of the checklist is shown in Table 1. The median overall score was 77.1%; ‘health education’ and ‘cold chain’ were the dimensions with the highest medians, while ‘special immunobiological agents’ and ‘general organization’ obtained the lowest medians.

**Table 1:** Description of the median, interquartile range and minimum and maximum values of scores, both overall score and by dimensions, of selected Brazilian vaccination rooms (n = 25), 2019

Dimensions of vaccination	Median	Interquartile range	Minimmum value	Maximmum value
Overall	77.1	66.4 - 81.7	43.5	89.3
General organization	58.3	41.7 - 66.7	25.0	75.0
General aspects of vaccination rooms	82.6	65.2 - 91.3	47.8	95.7
Technical procedures	78.6	64.3 - 85.7	50.0	100.0
Cold chain	86.7	80.0 - 90.0	43.3	96.7
Information system	71.4	57.1 - 80.9	23.8	100.0
Adverse events following vaccination	80.0	60.0 - 100.0	20.0	100.0
Special immunobiological agents	50.0	25.0 - 100.0	0.0	100.0
Epidemiological surveillance	75.0	75.0 - 100.0	25.0	100.0
Health education	100.0	75.0 - 100.0	0.0	100.0

A detailed analysis of the items revealed that, in the dimension related to ‘general organization’, in 20 vaccination rooms, the implementation or updating of standard operating procedure was not identified, and in 17, it was found that the team responsible for the vaccination room was unaware of such procedures. In addition, the lack of periodic training for professionals working in vaccination rooms was reported by those who were responsible for 15 rooms.

Regarding the dimension ‘special immunobiological agents’, in most vaccination rooms, the professionals were aware of the existing reference centers (n = 22) and the flow to request these immunobiological agents and/or referral to these services (n = 13). However, in 15 rooms, the professionals were unaware of the list of immunobiological agents available; and in 16, they were unaware of their indications.

**Figure 1:**
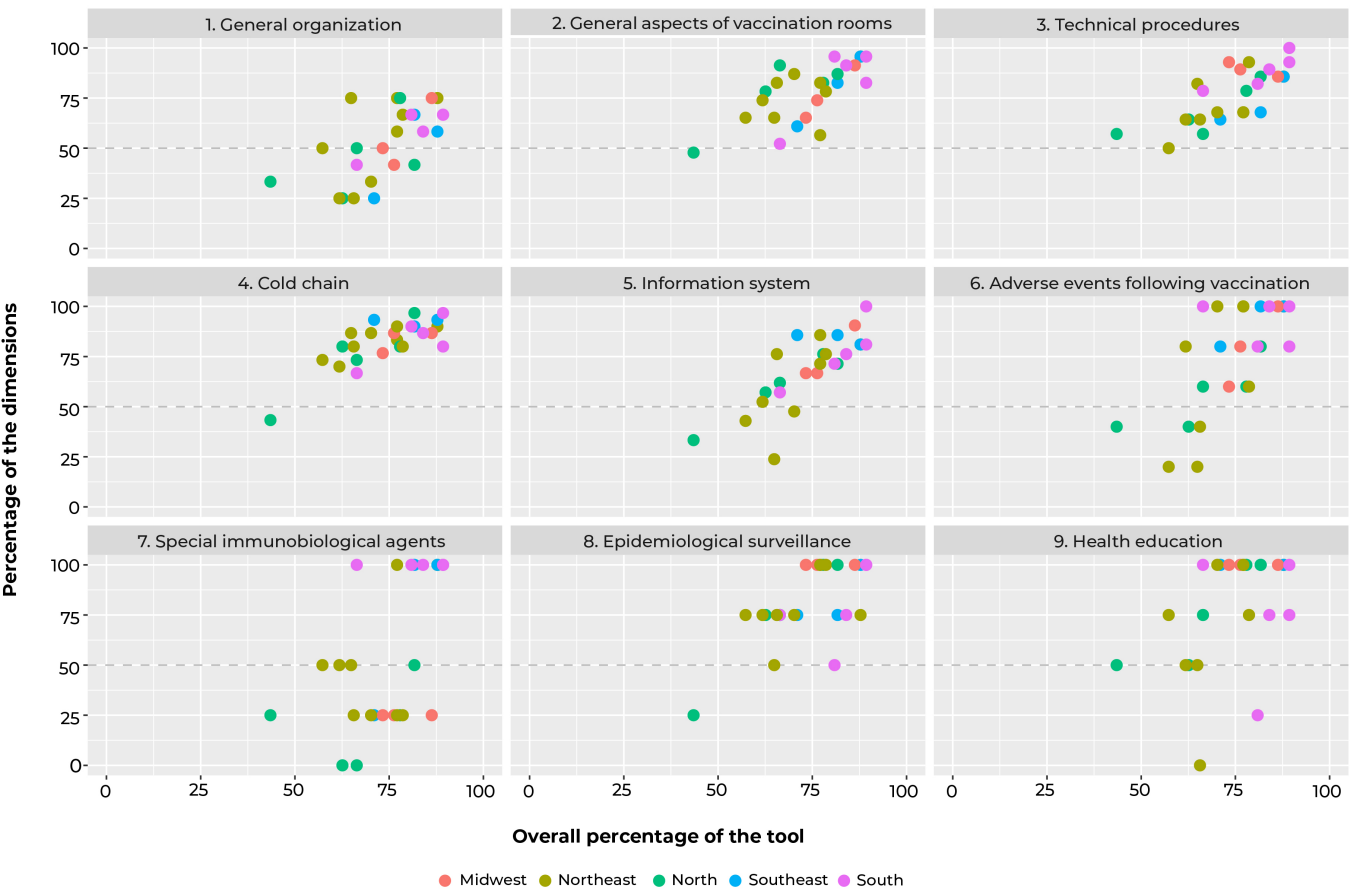
Distribution of scores, both overall and by dimension, of selected Brazilian vaccination rooms (n = 25), according to the macro-region where they are located, 2019

Figure 1 shows the distribution of scores, both overall and by dimensions, according to the location of the rooms in Brazilian macro-regions. It could be seen inter- and intra-regional heterogeneity, especially regarding the dimensions ‘information system’, ‘adverse events following vaccination’ and ‘special immunobiological agents’. Among the rooms with scores below 50% in the dimensions evaluated, a higher frequency of those located in the North and Northeast regions was found.

## Discussion

This study allowed us to observe a positive scenario in the general diagnosis of PHC vaccination rooms in several regions of the country, with emphasis on health education actions, communication of the importance of vaccination, and structural and logistical aspects of the cold chain. It is worth highlighting the need to prioritize the strengthening of actions related to the knowledge of professionals about special immunobiological agents and the general organization of rooms. Furthermore, inter-regional and intra-regional variability in the diagnosis of vaccination rooms was observed, with less favorable results for the North and Northeast regions.

The limitations of this study include the fact that the sample studied is not representative of Brazil and its regions, given that the allocation of the number of centers per region was not proportional (selection bias). Another limitation is related to the variable number of items per dimension of the checklist, which may weaken the comparability of percentage scores of the dimensions.

It is worth mentioning the need to update the tool, in line with the ‘Manual of Rules and Procedures for Vaccination’ of the Ministry of Health,^12^ and to standardize the evaluation criteria.

Based on the diagnosis of vaccination rooms, health education actions have stood out, favorable in most of them, a result similar to those of studies conducted in Minas Gerais^15^ and São Paulo.^16^ On the other hand, a study carried out in Pernambuco,^14^ when jointly evaluating the dimensions of health education and epidemiological surveillance, found unfavorable results. Thus, it is worth highlighting the need for the sustainability of educational actions, given that communication with the population make clear the importance of vaccines as individual and collective protective measures, in addition to demystifying rumors, contributing to the link between population and service, greater adherence to vaccination and maintenance of satisfactory vaccination coverage.^9^ ^,^ ^10^

Another dimension of great prominence that has been observed in this study and in the literature^14^ ^-^ ^17^ was the cold chain, with a satisfactory result and low variability among the services, possibly because they are items that have the power to directly affect the quality and safety of these supplies, despite possible losses due to irregularities in the conditioning and/or logistics of resources.^12^

It could be seen opportunities for improvement related to the dimension general organization, in the aspects of human resources, process standardization and training. The identification of shortage of human resources can lead to accumulation of tasks and weaknesses in communication and active search for users.^20^ Taking into consideration the constant updates of the immunization schedule and the variability in the professionals’ understanding of the service process, facts that have already been reported in other studies,^13^ ^,^ ^20^ it is worth highlighting the need for permanent education^21^ and standardization of procedures, aiming at an immediate response to the occurrence of vaccine-preventable diseases, such as the COVID-19 pandemic.

Another opportunity for improvement that should be prioritized, among the dimensions of the checklist, is related to the dimension special immunobiological agents, corroborating other studies.^15^ ^,^ ^17^ The fragility of health professionals’ understanding of special immunobiological agents may impair access to this type of input and, consequently, the quality of life of specific populations that need it.^22^ ^,^ ^23^ However, a study conducted in a municipality of São Paulo achieved satisfactory results for the dimension special immunobiological agents.^16^

The inter- and intra-regional inequalities observed in the diagnosis of vaccination rooms, with a less favorable result for most of the dimensions in the rooms in the North and Northeast regions, compared to those in the other regions, reflect the historical inequalities of investment in material and human resources in the Brazilian territory, and trends of a lower vaccination coverage in both regions aforementioned.^2^ ^-^ ^4^

This study expands the diagnosis of vaccination rooms in several macro-regions of Brazil, from a contemporary perspective, given that the literature on the subject is scarce and it is not recent. It is noteworthy that vaccination is part of the basic microprocesses of Primary Health Care,^24^ which should be implemented and monitored regularly in order to ensure conditions for the provision of quality services. Thus, the adopted checklist proved to be easily applicable in different contexts, and can serve as an example for the institutionalization of monitoring culture, evaluation and continuous improvement of immunization services.

Taking these results, it can be concluded that the diagnosis performed enabled the identification of opportunities for improvement in vaccination rooms, and that continuous monitoring and evaluation may support a more assertive action plan in different types of governance
